# Photoresponsive Activity of the Zn_0.94_Er_0.02_Cr_0.04_O Compound with Hemisphere-like Structure Obtained by Co-Precipitation

**DOI:** 10.3390/ma16041446

**Published:** 2023-02-09

**Authors:** Robson França, Francisca Pereira Araujo, Luan Neves, Arthur Melo, Alexsandro Lins, Adriano Santana Soares, Josy Anteveli Osajima, Yuset Guerra, Luciano Costa Almeida, Ramón Raudel Peña-Garcia

**Affiliations:** 1 Programa de Pós-Graduação em Engenharia Física, Unidade Acadêmica do Cabo de Santo Agostinho, Universidade Federal Rural de Pernambuco, Cabo de Santo Agostinho 52171-900, PE, Brazil; 2 Unidade Acadêmica do Cabo de Santo Agostinho, Universidade Federal Rural de Pernambuco, Cabo de Santo Agostinho 52171-900, PE, Brazil; 3 Programa de Pós-Graduação em Ciências e Engenharia dos Materiais, Universidade Federal de Piauí, Teresina 64049-550, PI, Brazil; 4 Departamento de Física, Universidade Federal do Piauí, Teresina 64049-550, PI, Brazil; 5 Departamento de Engenharia Química, Universidade Federal de Pernambuco, Recife 50670-901, PE, Brazil

**Keywords:** ZnO, rare-earth, transition metal, hemisphere-like structure, photocatalysis

## Abstract

In this work, a ZnO hemisphere-like structure co-doped with Er and Cr was obtained by the co-precipitation method for photocatalytic applications. The dopant’s effect on the ZnO lattice was investigated using X-ray diffraction, Raman, photoluminescence, UV-Vis and scanning electron microscopy/energy dispersive spectroscopy techniques. The photocatalytic response of the material was analyzed using methylene blue (MB) as the model pollutant under UV irradiation. The wurtzite structure of the Zn_0.94_Er_0.02_Cr_0.04_O compound presented distortions in the lattice due to the difference between the ionic radii of the Cr^3+^, Er^3+^ and Zn^2+^ cations. Oxygen vacancy defects were predominant, and the energy competition of the dopants interfered in the band gap energy of the material. In the photocatalytic test, the MB degradation rate was 42.3%. However, using optimized H_2_O_2_ concentration, the dye removal capacity reached 90.1%. Inhibitor tests showed that ^•^OH radicals were the main species involved in MB degradation that occurred without the formation of toxic intermediates, as demonstrated in the ecotoxicity assays in *Artemia salina*. In short, the co-doping with Er and Cr proved to be an efficient strategy to obtain new materials for environmental remediation.

## 1. Introduction

The population growth and increased demand for consumer goods, associated with inefficient waste management, have led to the accumulation of pollutants in water resources [1]. This is very worrying because recent research estimates that by 2050 around 65% of Earth’s population will face a shortage of drinking water [2]. Among the anthropic activities that generate waste, intense industrial activity has stood out for releasing recalcitrant compounds, such as dyes and herbicides, which pose risks to the ecosystem and human health [3,4]. These pollutants have complex carbon chains that are resistant to degradation by the environment and are difficult to remove in conventional water treatments [5].

Advanced oxidative processes (AOPs) can satisfactorily break down molecules of organic pollutants. The high efficiency of AOPs is related to the formation of oxygen radical species that are directly responsible for the degradation of the contaminant [6,7]. Heterogeneous photocatalysis is an example of promising AOPs because it is a simple, efficient and low-cost method and can be used to degrade different toxic compounds in wastewater [5,8]. In this technique, a semiconductor is used which, upon interaction with light, destabilizes the target molecules. When the semiconductor absorbs radiation, the valence band electron jumps to the conduction band, generating an electron–hole pair. This pair is responsible for the formation of important oxygenated radicals, mainly hydroxyl radicals, which attack the pollutant molecule [9,10,11].

Zinc oxide (ZnO) is a semiconductor that has been investigated for water purification, due to its physical and chemical stability, non-toxicity high sensitivity and wide band gap [12,13,14,15]. However, the rapid recombination of the electron–hole pair in ZnO strongly influences the photocatalytic activity [16,17]. Studies have shown that the inclusion of metal ions into the ZnO crystalline structure can delay the recombination of the electron–hole pair and contribute to the photocatalytic activity of this material [17,18,19,20]. Different studies proved that ZnO doped with transition metal ions presents a satisfactory photocatalytic response in recalcitrant compounds. Some of these studies included the addition of metals such as Co [21], Ag [22], Ni [23], Cu [24] and Mn [25]. The Cr insertion in the ZnO matrix is not a complicated process due to the proximity of the ionic radii of Cr^3+^ (0.62 Å) and Zn^2+^ (0.72 Å), and it can also be considered as an alternative to improve the photocatalytic response of ZnO. In general, Cr doping can promote the inhibition of the recombination of photogenerated electron–hole pairs [17]. In a classic photocatalysis experiment, Al Kalas et al. (2022) investigated the effect of different concentrations of Cr^3+^ ions on the structural, optical and photocatalytic properties of ZnO. The authors observed that the presence of the dopant in the ZnO lattice reinforced the oxygen defects that act directly to prevent the recombination of photogenerated charge carriers on the semiconductor surface. A better photocatalytic response was observed for the materials with low dopant concentrations [26]. Truong et al. (2019) also reported that Cr doping affected the band gap energy, expanding the semiconductor’s energy absorption into the visible region. This resulted in a material that was efficient in removing the pollutant methyl orange [17].

There are also some studies that reported doping with rare earth metals as a strategy to increase the photocatalytic response of ZnO. Beyond increasing the lifetime of charge carriers in the valence and conduction bands, doping with these metals can also cause photoactivation with visible light [27,28,29]. Sowik et al. (2018) investigated the effect of different types of rare earth metal (Eu, Ho, Tb, Yb, Er and La) in the degradation of organic pollutants under UV-Vis irradiation. Among the materials obtained, the sample containing Er^3+^ ions demonstrated the highest efficiency of contaminant removal (almost 90%), due to effects such as the decrease in the band gap energy and recombination of the electron–hole pair [30]. Pascariu et al. (2019) also explored the effect of rare earths on the structural, optical and photocatalytic properties of ZnO nanostructures. According to this study, all materials presented a dye removal capacity above 60% within UV irradiation. However, Er^3+^ and Sm^3+^ dopants induced greater increases in the photocatalytic performance of ZnO [31].

Co-doping processes have been used as a new strategic approach to improve the photocatalytic capacity of semiconductor materials [32,33,34]. In this sense, the present work aimed to investigate the effect of Er^3+^ and Cr^3+^ cations’ simultaneous addition on the structural, morphological and optical properties, as well as in the photocatalytic response of the ZnO hemisphere-like structure synthetized by co-precipitation. Specifically, we focused on the synthesis of the Zn_0.94_Er_0.02_Cr_0.04_O compound. The cations’ concentration (2% of Er^3+^ and 4% of Cr^3+^) in the ZnO host matrix was carefully determined to ensure that secondary phases were not formed. In addition, the photocatalytic response was investigated using the model pollutant methylene blue, a cationic dye that is resistant to biodegradation, representing risks to the ecosystem. Finally, the toxicity of the MB solution was investigated through ecotoxicity tests using *Artemia salina*.

## 2. Materials and Methods

### 2.1. Material Preparation 

For the Zn_0.94_Er_0.02_Cr_0.04_O compound synthesis, the following reagents were used: zinc acetate (Aldrich, 98.0%, Barueri, Brazil), Erbium nitrate (Aldrich, 99.0%), Chromium nitrate (Aldrich, 99.0%) and distilled water as the solvent. Desired amounts of raw materials were weighed and dissolved in 100 mL of distilled water by magnetic stirring for 1 h. Then, the pH of the system was adjusted (pH = 9) by dropping a NaOH solution, and it was kept under constant stirring for 1 h until precipitate formation. The solid was washed five times with distilled water and then three times with ethanol. The material was dried at 100 °C (heating ramp 1 °C/min) for 6 h.

### 2.2. Characterization

The compound was characterized by X-ray diffraction (XRD) using an X-ray diffractometer, model D8 Advance from Bruker with Cu-Kα radiation (λ = 1.5406 Å), in the Bragg-Betano geometry. The Raman spectrum was measured with a Raman spectrometer, model Santerra from Bruker (Billerica, MA, USA), with an Olympus BX50 microscope coupled (Tokyo, Japan). The photoluminescence spectrum was measured in a Spectrofluorometer Horiba-JobinYvon Fluorolog-3 (Kyoto, Japan), while the diffuse reflectance spectra was obtained on a UV-Vis spectrometer, Shimadzu, UV-2700 (Kyoto, Japan). The spectra were deconvoluted using Gaussian fit. The morphology was obtained in a scanning electron microscope (SEM), and a TESCAN MIRA3 model (Brno, Czech Republic) with an energy dispersive X-ray spectrometer (EDS) coupled.

### 2.3. Photocatalytic Test

Photocatalytic tests were performed into a borosilicate reactor using a commercial lamp (160 W) as a source of UV radiation. In each of the tests, the photocatalyst concentration was 0.5 g·L^−1^ and methylene blue (MB) dye solution (1.0 × 10^−5^ mol·L^−1^) was used as a model pollutant. The tests were performed at 25.0 ± 1.0 °C under magnetic stirring. The radiation intensity was monitored using a radiometer (Hanna). The adsorption equilibrium was reached in 30 min in the dark. Aliquots irradiated were removed during the test and centrifuged at 4400× *g* rpm for 45 s twice. The band at 664 nm was used to monitor the MB dye degradation using a spectrophotometer (Agilent Technologies, Cary 60 UV–Vis) in the range between 200 and 800 nm. The MB degradation was calculated by Equation (1).

(1) $$Degradation\left(\%\right)=\frac{C_{0}-C}{C_{0}}\times 100$$

where *C*_0_ and *C* correspond to the initial dye concentration and the final dye concentration (t = 150 min), respectively. The photocatalytic systems were also investigated using hydrogen peroxide (100 ppm) under optimal concentration [35]. To identify the roles of different reactive species, radical scavenger studies were performed using the ethylenediaminetetraacetic acid (EDTA) (2.4 × 10^−6^ mol L^−1^), methyl alcohol (3.4 × 10^−3^ mol L^−1^) and silver nitrate (5.0 × 10^−4^ mol L^−1^), which are inhibitors of hole (h^+^), hydroxyl radical (^•^OH) and electrons (e^−^), respectively. The reusability of the material was tested for up to three consecutive cycles and the degradation rate determined in each case.

### 2.4. Ecotoxicity

The toxicity of the irradiated solution with photocatalyst material was analyzed using *Artemia salina* microcrustaceans. The nauplii were cultivated in a synthetic saline solution with constant illumination for 48 h. After this time, the nauplii were added to the solution containing MB dye-irradiated and synthetic saline solution (1:1 *v*/*v*). The survival rate of *Artemia salina* was evaluated after 24 h and 48 h [32].

## 3. Results and Discussion

### 3.1. Structural, Optical and Morphological Characterization

**Figure 1:**
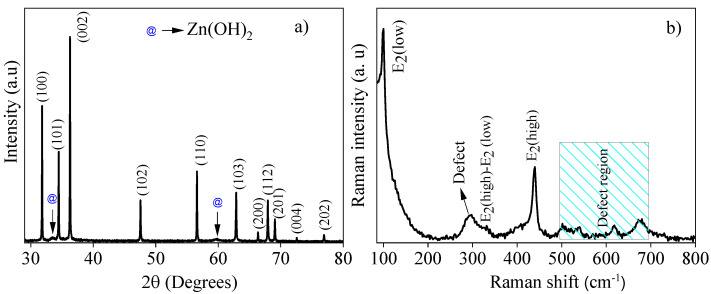
(**a**) X-ray diffraction pattern; (**b**) Raman spectrum for the Zn_0.94_Er_0.02_Cr_0.04_O compound.

Figure 1 presents the X-ray diffraction pattern and Raman spectrum for the Zn_0.94_Er_0.02_Cr_0.04_O compound synthesized via co-precipitation. In Figure 1a, we can see that the compound presents diffraction peaks representative of the ZnO wurtzite structure with P6_3_mc space group, which are identified by the crystallographic card JCPDS No. 36-1451. In addition, there are two diffraction peaks at 2θ values of 33.5° and 59.5° marked with (@) which are indexed to the Zn(OH)_2_ phase with data from the crystallographic card: JCPDS No. (38-0356). The secondary phase formation may be a consequence of the calcination temperature used in this study (100 °C for 6 h).

The lattice parameters (*a* and *c*) are calculated using the equation that correlates the interplanar spacing, Miller indices and the lattice constants for the ZnO with hexagonal structure [36]. The calculated values for *a* and *c* are 3.2468(1) Å and 4.9482(3) Å, respectively, which are smaller than the recently reported values for ZnO [37]. This indicates that the dopants’ insertion into the ZnO host lattice produces structural distortions that can be associated with the difference between the ionic radii of the dopant cations (Er^3+^ (0.89 Å) and Cr^3+^ (0.62 Å)) and the Zn^2+^ (0.74Å) host cations [38].

The Scherrer equation [36] was used to estimate the average crystallite size (D) and the value obtained was 86 nm. This result is four times higher than the value reported for ZnO nanostructures by Rocha et al. [37]. The Er and Cr ions’ insertion in the ZnO structure can be classified as the donor’s dopants because they have more of an oxidation state, if compared to the Zn ions. This difference can cause imperfections in the ZnO structure or attenuate the present interstitial defects; as defects are essential for structural changes, an increase in the average crystallite size could happen as Zn cations are replaced by Er and Cr cations. Furthermore, the energetic competition between the dopant cations, because they have different characteristics (ionic radii, electronegativity, etc.), may contribute to the nucleation and growth mechanisms, increasing the driving force that contributes to the crystallite’s growth [36,39,40].

Figure 1b exhibits the room temperature Raman spectrum for the Zn_0.94_Er_0.02_Cr_0.04_O compound. For ZnO with the hexagonal wurtzite structure, the optical mode frequencies of the Brillouin zone are in the region between 99 and 600 cm^−1^ [41,42,43,44,45]. The E_2_^(low)^ and B_1_^(low)^ modes emerge in the low frequency regions (100 cm^−1^ and 275 cm^−1^, respectively); the E_2_^(high)^, E_1_(TO) and A_1_(TO) are found at frequency zones between 370 cm^−1^ and 440 cm^−1^; while for higher frequencies (500 cm^−1^ to 700 cm^−1^) the E_1_(LO), A_1_(LO) and B_1_^(high)^ modes are more common [41,42,43,44,45]. For the compound under study, the E_2_^(Low)^ mode is present at the frequency of 99 cm^−1^, the E_2_^(high)^ − E_2_^(low)^ modes emerge as a shoulder at 331 cm^−1^ while an intense peak located at 437 cm^−1^ belongs to the E_2_^(high)^ mode [43,45]. In Figure 1b, a broad peak centered at 294 cm^−1^ is noted. According to the literature, the B_1_^(low)^ mode emerges at approximately 275 cm^−1^ and is related to the structural defects of Zn atoms located in neighboring interstitial sites and oxygen vacancies [41,42,43,44,45]. The shift to higher frequencies (294 cm^−1^) can be attributed to an increasing number of defects due to the Er and Cr cations’ inclusion in the structure, since they have distinct characteristics in relation to Zn cations (Er^3+^ (0.89 Å), Cr^3+^ (0.62 Å) and Zn^2+^ (0.74Å)) [38]. For the region between 500 and 700 cm^−1^, the emergence of E_1_(LO), A_1_(LO) and B_1_^(high)^ modes has been reported [44,45]. In the present study, peaks appear at 501, 539, 620 and 678 cm^−1^, which may be associated with oxygen vacancy (V_o_) defects that arise from the difference between the oxidation states of the dopant cations and the host cation [44,45]. This conclusion is also based on the results reported by Rocha et al. [37] for pure ZnO structures, since for the region marked in Figure 1b (defect region), the authors did not observe characteristic peaks of the optical mode frequencies of the Brillouin zone for the wurtzite structure.

**Figure 2:**
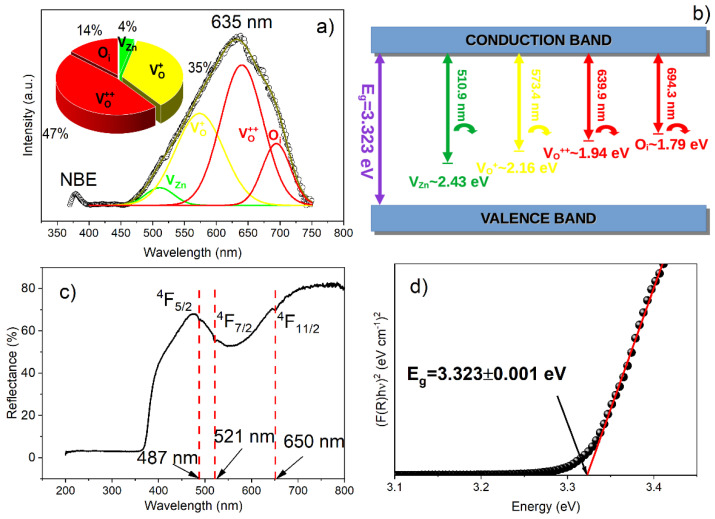
(**a**) PL spectrum at room temperature, (**b**) PL spectrum deconvolution, (**c**) diffuse reflectance spectrum and (**d**) optical band gap. The inset of (**b**) displays the pie charts of the relative percentage of defects for the Zn_0.94_Er_0.02_Cr_0.04_O compound.

To attest the defects emerging from the dopant’s inclusion, the PL spectrum of the Zn_0.94_Er_0.02_Cr_0.04_O compound is measured at room temperature in the 370–750 nm range and excitation of 340 nm (Figure 2a). In Figure 2a, two well-defined peaks are observed; the first, located in the UV region and centered at 379 nm, is typical of the near-band emission (NBE) of ZnO and is related to excitonic recombination in the band gap region (between the valence and conduction bands), and the second wide peak in the visible region that is centered at 635 nm is characteristic of intrinsic defects of the lattice [46,47,48,49,50,51]. The form of the peak located in the visible region confirms the degree of point defects that emerge in the compound due to the dopant’s inclusion, synthesis route, pH, annealing temperature, etc. [38]. In the present study, the difference in the oxidation state of the dopant cations and the host cations, as well as their ionic radius, play the key role for the development of high defects’ concentrations in the structure.

To quantify the point defects present in the hexagonal structure after the Er and Cr cations’ inclusion, the PL spectrum is deconvolved using a Gaussian distribution function. The defects of V_o_ are related to the origin of green emission; in addition, it has been shown that V_Zn_ also contributes to broadband emission in the green region [46,52,53]. In our sample, the green emission centered at ~510.9 nm (2.43 eV) corresponds to V_Zn_. The bands related to V_o_ defects appear in higher wavelength regions. Commonly, the V_o_ defects reported for ZnO nanostructures appear in three states, i.e., neutral oxygen vacancy (V_o_), single charge (V_o_^+^) and double charge (V_o_^++^) [53]. Note that, in our sample, there are no defects associated with neutral oxygen vacancies (V_o_). The yellow emission at ~573.4 nm (2.16 eV) originates from V_o_^+^ and the orange-red emission at ~639.9 nm (1.94 eV) derives from V_o_^++^. Finally, the peak centered in the red region at ~694.3 nm (1.79 eV) can be attributed to O_i_ [37]. The simplified scheme of the transitions from the conduction band to the valence band is represented in Figure 2b. The defects’ quantification is shown in the pie chart, presented in the inset of Figure 2a, which contains the percentage of the area under the Gaussian curve and shows the relative concentration of point defects existing in the Zn_0.94_Er_0.02_Cr_0.04_O compound. 

Note that the simply ionized (V_o_^+^ (35%)) and double ionized (V_o_^++^ (47%)) oxygen vacancies’ defects are predominant in the compound, which is evident because divalent Zn^2+^ cations are substituted by trivalent Er^3+^ and Cr^3+^ cations. In addition, other defects that appear in the sample, but in lower concentrations, are oxygen interstitial (O_i_ (14%)) and zinc vacancy (V_Zn_ (4%)). Similar results have been reported in the literature for rare earth and transition metal-doped ZnO compounds [46,47,48,49,50,51]. On the other hand, the UV–Vis diffuse reflectance spectrum of the Zn_0.94_Er_0.02_Cr_0.04_O compound is shown in Figure 2c. We can see three defined bands at 487, 521 and 650 nm, which certify the replacement of Zn cations by Er cations in the ZnO crystal structure. According to the literature, these bands point to the transitions from the ground level (^4^I_15/2_) to the excited states (^4^F_5/2_), (^4^F_7/2_) and (^4^F_9/2_) when dopant Er cations are inserted into the hexagonal structure [54]. Using the Kubelka–Munk model and Tauc’s plot, the band gap energy of the compound is estimated (Figure 2d) [55]. The calculated value (3.323 ± 0.001 eV) is in agreement with the value reported for ZnO bulk (3.35 eV) [49]. However, a comparison with the value reported by Rocha et al. [37] indicates that the Er and Cr inclusion in the ZnO host lattice can cause an energetic competition, which allows the Fermi level to move for the conduction band, increasing the concentration of carriers and causing an increase in the optical gap [56]. Similar results have been observed by different authors for doped ZnO structures [49,56].

**Figure 3:**
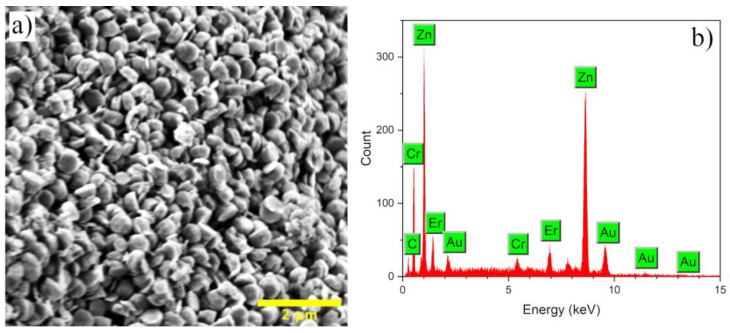
(**a**) SEM image and (**b**) EDS spectrum of the Zn_0.94_Er_0.02_Cr_0.04_O compound synthetized by co-precipitation.

The morphology of the Zn_0.94_Er_0.02_Cr_0.04_O compound was studied by SEM and the image is shown in Figure 3a. As observed, the sample presents agglomerates of hemisphere-like particles with sizes between 80 to 100 nm, being in the order of the crystallite sizes calculated from the Scherrer equation (86 nm).

Different morphologies have been obtained for the ZnO compound (flowers, rods, disks, hexagons, etc.), which are highly reliant on pH, temperature and synthesis methods [32,57,58,59]; however, hemisphere-like structures are not very common, which leads us to assume that there is a solid influence of Er^3+^ and Cr^3+^ cations on the ZnO morphology, possibly favoring the nucleation and growth mechanisms. Finally, Figure 3b shows the EDS spectrum of the compound Zn_0.94_Er_0.02_Cr_0.04_O, where characteristic peaks of the Zn, O, Er and Cr elements can be observed, which certify the hexagonal structure formation, as well as the replacement of Zn^2+^ cations by Er^3+^ and Cr^3+^ cations in the host lattice. The peaks of Au and C elements are characteristic of the sample coating and sample holder, respectively.

### 3.2. Photocatalytic Performance

**Figure 4:**
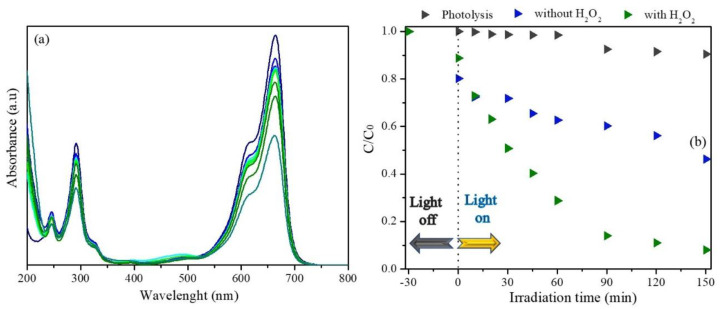
Photocatalytic study of Zn_0.94_Er_0.02_Cr_0.04_O sample: (**a**) spectral variation and (**b**) *C*/*C*_0_ ratio comparative of the photocatalytic system with and without H_2_O_2_ as a function of irradiation time.

The photocatalytic response of the Zn_0.94_Er_0.02_Cr_0.04_O using an MB dye contaminant is presented in Figure 4. According to the absorption spectra (Figure 4a), the absorption maximum at 664 nm decreases during system irradiation, that can be attributed to dye degradation reactions mediated by the material synthesized. In addition, the increase in the absorption in the region between 400 and 525 nm is associated to the formation of photoproducts that absorb in this region [57,60,61]. After 150 min, the MB degradation rate is found to be 42.3%. On the other hand, in the photolysis test, the MB degradation is ~10.60%.

To optimize the photocatalytic response of the Zn_0.94_Er_0.02_Cr_0.04_O material, 100 ppm at H_2_O_2_ is added to the reaction medium. This concentration is used based on previous descriptions in the literature that consider it adequate for semiconductor ZnO photocatalytic processes [35]. In this condition, the dye removal capacity is 90.1%. The addition of H_2_O_2_ in the heterogeneous photocatalysis increases the organic pollutant degradation rate, because H_2_O_2_ can capture the photogenerated electrons efficiently and delay the recombination of the electron/hole pair [62].

**Figure 5:**
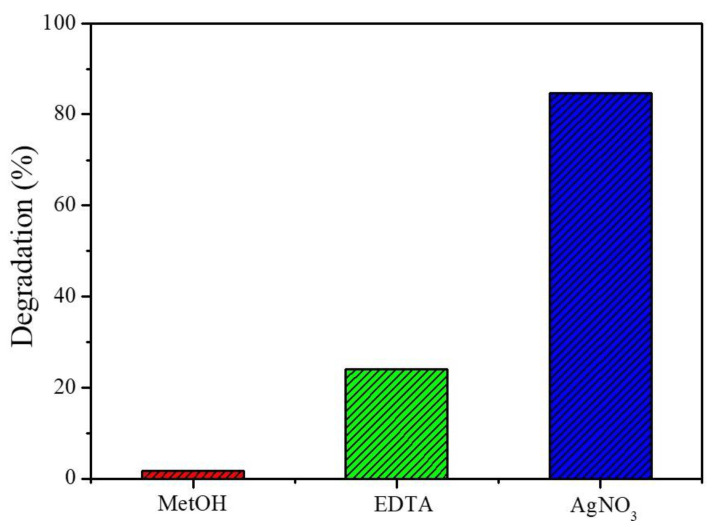
Photocatalytic systems containing different inhibitor reagents.

Scavenger tests are performed to determine the contribution of species involved in the degradation of MB by the Zn_0.94_Er_0.02_Cr_0.04_O photocatalyst, and the results are displayed in Figure 5. The degradation rate of MB is 1.7%, 24.0% and 84.8% in tests using MetOH, EDTA and AgNO_3_ as scavengers, respectively. As seen, the MB removal is drastically affected when MetOH is added to the photocatalytic system. This reagent is well reported in the literature for capturing ^•^OH radicals in the photocatalytic medium. Thus, this result suggests that ^•^OH radicals are directly involved in the photocatalytic activity of Zn_0.94_Er_0.02_Cr_0.04_O. Using EDTA, that is a hole scavenger, the dye removal capacity also increases, indicating that these species also participate in the photocatalytic activity of the semiconductor.

**Figure 6:**
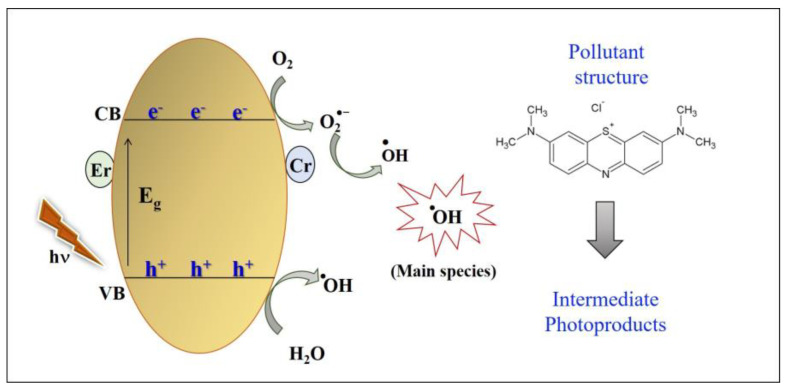
Photocatalytic mechanism of Zn_0.94_Er_0.02_Cr_0.04_O material.

Interestingly, the rate of dye degradation increases when AgNO_3_ is added to the reaction medium. This reagent captures electrons from the conduction band and the recombination of the e^−^/h^+^ pair is difficult, favoring the photocatalytic reactions. Sá et al. (2021) reported similar results and attributed them to the electron capture by Ag^0^ deposited on the semiconductor surface [63]. Based on these results, a brief mechanism involved in photocatalysis of the Zn_0.94_Er_0.02_Cr_0.04_O sample is illustrated in Figure 6. When the Zn_0.94_Er_0.02_Cr_0.04_O compound absorbs a photon of energy, the charge carrier’s electrons (e^−^) and holes (h^+^) are formed in the conduction (BC) and valence (VB) bands, respectively. These charges react with molecular species adsorbed on the semiconductor surface. Different reactions at the solid–liquid interface cause the formation of ^•^OH radicals that are important to react with the structure of the pollutant forming intermediate photoproducts.

**Figure 7:**
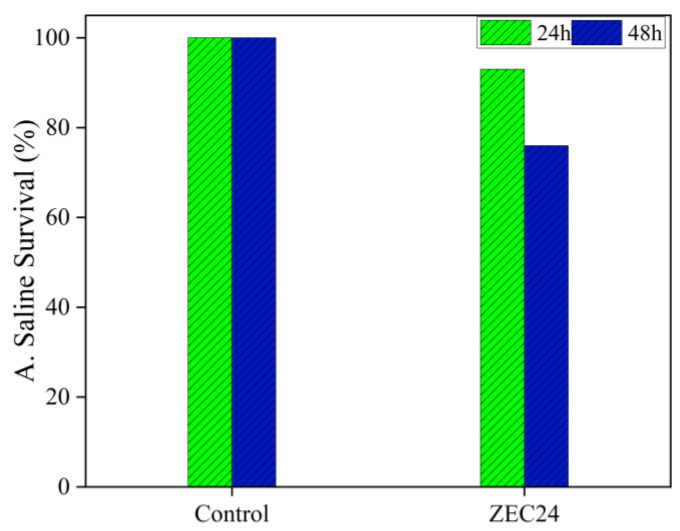
Ecotoxicity essay using *Artemia salina*.

The MB dye solution irradiated with the sample obtained in this study is investigated from the ecotoxicity assay using *Artemia salina*. It is known that during the photodegradation of dyes, the intermediates formed can be more toxic than the dye itself [64]. As *A. salina* are microcrustaceans sensitive to the presence of contaminants, the test is efficient to verify the toxicity of intermediates formed in the photocatalytic process [65]. The results are expressed in terms of nauplii survival after 24 and 48 h of contact with the irradiated solution and are shown in Figure 7. Observing the results, at both investigation times, the survival rate of microcrustaceans is lower than the control group. However, these values are greater than 50%, indicating that the intermediate photoproducts are not toxic [66].

**Figure 8:**
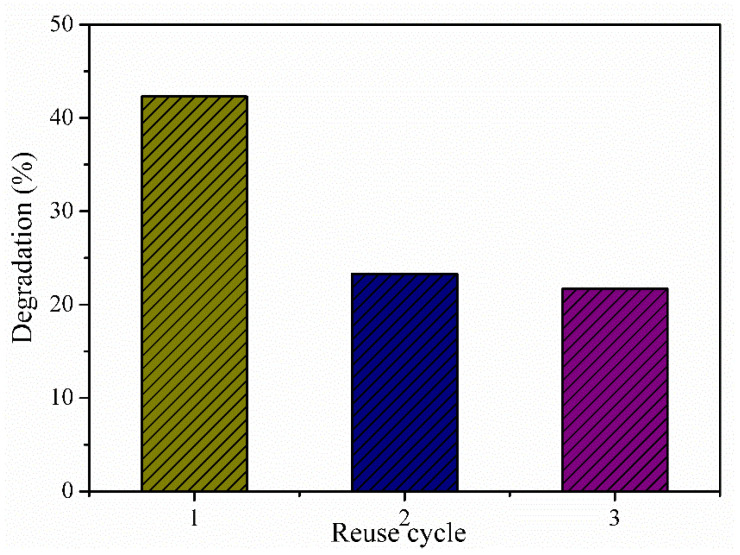
Reuse of Zn_0.94_Er_0.02_Cr_0.04_O for photocatalysis in three consecutive cycles.

Photocatalyst recycling is considered for three consecutive cycles in this study. As shown in Figure 8, the MB removal capacity decreases after the second reuse, in which a degradation rate of 23.3% is observed. In the third reuse, the photocatalytic response of the material is 21.3%. The decrease in the rate of degradation in reuse cycles can occur due to mass loss and the photocorrosion process of the ZnO when subjected to UV radiation [14,32].

## 4. Conclusions

The Zn_0.94_Er_0.02_Cr_0.04_O compound with a hemisphere-like structure was successfully obtained using the co-precipitation method. Secondary phases of the dopant cations were not identified, suggesting that the concentrations of dopants used did not exceed the solubility limit in the ZnO lattice. The predominance of oxygen vacancies confirmed by the PL spectrum deconvolution suggests that dopant cations occupied the position of the Zn cations in the ZnO crystal structure. However, the differences between the ionic radii of the dopant species, in relation to the Zn cation, caused a lattice contraction. MB removal occurred due to mechanisms involving ^•^OH radicals. These species are very efficient to degrade organic molecules, generating non-toxic photoproducts. Under optimized conditions, it is possible to potentiate the photocatalytic activity of Zn_0.94_Er_0.02_Cr_0.04_O. Finally, this study suggests that the ZnO co-doped with Er^3+^ and Cr^3+^ can be strategically beneficial for removing pollutants via photocatalysis.

## Data Availability

Not applicable.
